# Metabolic landscape and pathogenic insights: a comprehensive analysis of high ovarian response in infertile women undergoing in vitro fertilization

**DOI:** 10.1186/s13048-024-01411-6

**Published:** 2024-05-17

**Authors:** Ling-Ling Ruan, Xing-Yu Lv, Yu-Lin Hu, Ming-Xing Chen, Jing-Tang, Zhao-Hui Zhong, Mei-Hua Bao, Li-Juan Fu, Xin Luo, Shao-Min Yu, Qi Wan, Yu-Bin Ding

**Affiliations:** 1 Department of Obstetrics and Gynecology, Women and Children’s Hospital of Chongqing Medical University, No. 23 Central Park North Road, Yubei District, Chongqing, 401147 PR China; 2 Joint International Research Laboratory of Reproduction and Development of the Ministry of Education of China, School of Public Health, Chongqing Medical University, Chongqing, 400016 China; 3 Department of Obstetrics and Gynecology, the People’s Hospital of Yubei District, No. 23 Central Park North Road, Chongqing, 401120 China; 4 Department of Obstetrics and Gynecology, West China Second Hospital, Sichuan University, Chengdu, 610041 China; 5 Key Laboratory of Birth Defects and Related Diseases of Women and Children (Sichuan University), Ministry of Education, Chengdu, 610041 China; 6 The Reproductive Center, Sichuan Jinxin Xinan Women & Children’s Hospital, Chengdu, Sichuan, 610011 China; 7 Department of Pharmacology, Academician Workstation, Changsha Medical University, Changsha, 410219 China; 8 Department of Obstetrics and Gynecology, the First Affiliated Hospital of Chongqing Medical University, Chongqing, 400016 China; 9 Department of Bioinformatics, School of Basic Medicine, Chongqing Medical University, Chongqing, 400016 People’s Republic of China

**Keywords:** High ovarian response, Polycystic ovary syndrome, Serum, Metabolome

## Abstract

**Background:**

In the realm of assisted reproduction, a subset of infertile patients demonstrates high ovarian response following controlled ovarian stimulation (COS), with approximately 29.7% facing the risk of Ovarian Hyperstimulation Syndrome (OHSS). Management of OHSS risk often necessitates embryo transfer cancellation, leading to delayed prospects of successful pregnancy and significant psychological distress. Regrettably, these patients have received limited research attention, particularly regarding their metabolic profile. In this study, we aim to utilize gas chromatography-mass spectrometry (GC-MS) to reveal these patients’ unique serum metabolic profiles and provide insights into the disease’s pathogenesis.

**Methods:**

We categorized 145 infertile women into two main groups: the CON infertility group from tubal infertility patients and the Polycystic Ovary Syndrome (PCOS) infertility group. Within these groups, we further subdivided them into four categories: patients with normal ovarian response (CON-NOR group), patients with high ovarian response and at risk for OHSS (CON-HOR group) within the CON group, as well as patients with normal ovarian response (PCOS-NOR group) and patients with high ovarian response and at risk for OHSS (PCOS-HOR group) within the PCOS group. Serum metabolic profiles were analyzed using GC-MS. The risk criteria for OHSS were: the number of developing follicles > 20, peak Estradiol (E2) > 4000pg/mL, and Anti-Müllerian Hormone (AMH) levels > 4.5ng/mL.

**Results:**

The serum metabolomics analysis revealed four different metabolites within the CON group and 14 within the PCOS group. Remarkably, 10-pentadecenoic acid emerged as a discernible risk metabolite for the CON-HOR, also found to be a differential metabolite between CON-NOR and PCOS groups. cysteine and 5-methoxytryptamine were also identified as risk metabolites for the PCOS-HOR. Furthermore, KEGG analysis unveiled significant enrichment of the aminoacyl-tRNA biosynthesis pathway among the metabolites differing between PCOS-NOR and PCOS-HOR.

**Conclusion:**

Our study highlights significant metabolite differences between patients with normal ovarian response and those with high ovarian response and at risk for OHSS within both the tubal infertility control group and PCOS infertility group. Importantly, we observe metabolic similarities between patients with PCOS and those with a high ovarian response but without PCOS, suggesting potential parallels in their underlying causes.

**Supplementary Information:**

The online version contains supplementary material available at 10.1186/s13048-024-01411-6.

## Introduction

The latest report from the World Health Organization (WHO) highlights a statistic: approximately 17.5% of adults globally, equivalent to one in six patients, grapple with infertility [1]. Assisted reproductive technology (ART) emerges as a potent solution to help this demographic achieve pregnancy [2]. At the core of ART lies controlled ovarian stimulation (COS), which employs ovulation-stimulating drugs to simultaneously foster the simultaneous development of multiple ovarian follicles, facilitating the retrieval of numerous mature oocytes [2]. However, the pursuit of a high ovarian response presents a formidable challenge: mitigating the risk of ovarian hyperstimulation syndrome (OHSS). This iatrogenic complication manifests as ovarian cystic enlargement, heightened vascular permeability, and fluid extravasation into the third space, potentially leading to severe consequences such as acute respiratory distress, anuria/acute renal failure, thromboembolism, etc [3]. Current estimates indicate the incidence of moderate to severe OHSS in patients undergoing in vitro fertilization (IVF) at 1-5% [4]. Although various strategies have been employed to reduce OHSS incidence, consensus remains elusive due to divergent efficacy and safety outcomes [4, 5].

Concurrently, when COS technology is employed to augment oocyte numbers, some patients may experience high ovarian response, with 29.7% of these patients being at high risk of developing OHSS (Supplementary Fig. 1). For this high-risk group of OHSS, clinicians will generally cancel embryo transfer to minimize the occurrence of late-onset OHSS in them [6, 7]. This decision not only prolongs the path to successful conception but also poses a mental and physical challenge for the patient. However, there has been insufficient research attention directed towards this specific group, as the majority of studies have predominantly focused on individuals already diagnosed with OHSS. Therefore, urgent investigation is warranted to delineate differences between these patients and those with normal ovarian response, elucidate the underlying physiological and pathological mechanisms, and provide clues for targeted care in the future.

Metabolomics has proven valuable in exploring disease etiology and understanding normal physiological conditions [8–10]. Recent studies have unveiled notable differences in maternal serum levels of niacin and niacinamide metabolic-pathway-related substances between women with poor ovarian response and control groups [11]. Simultaneously, changes in maternal serum glutamic acid, aspartate, and 1-methylnicotinamide levels were significantly associated with improved symptoms in women with PCOS [12]. In cases of OHSS symptoms, researchers identified elevated follicular fluid levels of mannitol and pyruvate alongside decreased levels of L-carnitine and creatinine [13]. Additionally, investigations into women undergoing IVF revealed correlations between the majority of amino acids in serum and the number of mature oocytes [14]. Despite these advancements, the metabolic profiles of patients who exhibit high ovarian response and are at risk for OHSS remain unknown.

Given the prevalence of OHSS risk among a significant number of patients with PCOS and the close association between PCOS and reproductive issues [15], it is imperative to investigate the pathogenic mechanisms within the PCOS population. This exploration is essential for enhancing OHSS incidence management and improving pregnancy success rates despite the apparent effectiveness of infertility treatments/strategies, including IVF as the final step [16]. Furthermore, while some studies have identified abnormal expression of unsaturated fatty acid metabolites in follicular fluid as a risk factor for OHSS in PCOS patients using metabolomics [17], it remains unclear whether the presence of abnormally expressed metabolites in maternal serum is also associated with high ovarian response in PCOS patients.

Considering the intricate connection among high ovarian response, PCOS, and OHSS, this study aims to investigate the serum metabolic profiles of patients who exhibit high ovarian response and are at risk for OHSS within the tubal infertility and PCOS framework. The objective is to elucidate the metabolic profiles of this frequently overlooked population, thereby offering a valuable reference point for further research on the pathological mechanisms.

## Materials and methods

### Participants

This study obtained ethical approval from both Sichuan Jinxin Xinan Women & Children’s Hospital (No. 2021014) and the Ethics Committee of Chongqing Medical University (No. 2021060). Informed consent was acquired from all participants, adhering to the principles outlined in the Declaration of Helsinki. The proportion of different ovarian responses following COS in ART was conducted utilizing data from 19,240 participants who underwent ART for the first time between January 2021 and December 2022. This data was sourced from the electronic database of Sichuan Jinxin Xinan Women & Children’s Hospital. The metabolomics investigation focused on participants from the “CYART Cohort,” a study group established at Sichuan Jinxin Xinan Women and Children**’**s Hospital in southwest China [18]. From December 2021 to December 2022, serum samples from 145 participants were selected for metabolic analysis based on specific inclusion and exclusion criteria, which are outlined as follows:

Inclusion criteria: (1) Ovulation-Stimulating Regimen: GnRH antagonist regimen. (2) Embryo Transfer Cycle Specification: the first embryo transfer cycle. (3) Age between 20 and 35 years, (4) Body Mass Index (BMI) ≤ 28 kg/m². (5) Infertility factors in the CON group: Tubal factors. (6) The diagnosis of PCOS is based on the “Chinese Polycystic Ovary Syndrome Diagnosis and Treatment Guide,” established by the Endocrinology Group of the Gynecology and Obstetrics Branch of the Chinese Medical Association in 2018 [19]. These diagnostic criteria are derived from the Rotterdam criteria [20].

Exclusion criteria: (1) History of ovarian surgery, including procedures such as cyst dissection and oophorectomy. (2) Hormone therapy within three months before treatment. (3) Contraindications to ovulation induction therapy. (4) Other systemic abnormalities, including genetic, endocrine, infectious and autoimmune diseases.

Subsequently, these 145 participants were categorized into two main groups based on clinical diagnosis: The control group (CON, *n* = 80) comprised patients clinically diagnosed with tubal factor infertility during IVF. The PCOS group (*n* = 65) consisted of patients clinically diagnosed with PCOS infertility during IVF. Within the CON group, we further subdivided them into two categories: The normal response group (CON-NOR, *n* = 40) comprised patients diagnosed with tubal infertility who exhibited a normal ovarian response during IVF (10–15 oocytes after COS). The high response group (CON-HOR, *n* = 40) comprised patients diagnosed with tubal infertility who exhibited a high ovarian response and were at risk for OHSS during IVF (> 15 oocytes after COS). Similarly, within the PCOS group, we subdivided them into two categories: The normal response group (PCOS-NOR, *n* = 26) comprised patients diagnosed with PCOS infertility who exhibited a normal ovarian response during IVF (10–15 oocytes after COS). The high response group (PCOS-HOR, *n* = 39) comprised patients diagnosed with PCOS infertility who exhibited a high ovarian response and were at risk for OHSS during IVF (> 15oocytes after COS). The risk criteria for OHSS were: the number of developing follicles > 20, peak Estradiol (E2) > 4000pg/mL, and Anti-Müllerian Hormone (AMH) levels > 4.5ng/mL.

### GnRH antagonist regimen

Between the second and fourth days of the menstrual period, gonadotropin (Gn) medications (such as Urofollitropin, Menotropins, Gonal F, Puregon, Fostimon, Menopur, Kim Sai Heng) were administered at a dosage ranging from 100 to 300 IU/day, with close monitoring of follicle development. Dosage adjustments to the Gn were made as necessary. Upon reaching a dominant follicle diameter of ≥ 12–14 mm or on gonadotropin days 5–6, daily subcutaneous injections of 0.25 mg of gonadotropin-releasing hormone (GnRH) antagonists (ganirelix acetate) were initiated and continued until the day of ovulation trigger.

The reference standards for triggering ovulation can be determined based on the size and quantity of target follicles and the levels of E2, LH, and progesterone. Ovulation triggering is initiated when there are three dominant follicles with a diameter of ≥ 17 mm each or two dominant follicles with a diameter of ≥ 18 mm each, while also considering the progesterone and estradiol levels. Two options are available for the triggering process: recombinant hCG at 250 µg or triptorelin acetate at 0.2 mg can be utilized.

### Serum collection

All serum samples were obtained on the day of the ovulation trigger. Fasting venous blood was carefully collected into tubes, followed by centrifugation at 3500 rpm for 10 min at 4 ℃. The isolated serum was then subpackaged into new EP tubes, rapidly frozen in liquid nitrogen, and stored in a -80 °C refrigerator for long-term preservation until further analysis.

### Serum sample preparation and derivatization for GC-MS analysis

Thawing was conducted in an ice bath to ensure the quality of serum samples. A 150 µl aliquot of serum was then mixed with 50 µl 4 M NaOH, 4 µl internal standard (10 mM 2,3,3,3-D4-alanine, Sigma, USA), and 200 µl methanol. After a 15-minute incubation at room temperature, the mixture underwent centrifugation at 12,000 rpm for 15 min. Following centrifugation, 300 µl of the supernatant was carefully transferred to a new glass test tube, where 34 µl pyridine (Sigma, USA) was added and thoroughly mixed. Next, 20 µl methyl chloroformate (Sigma, USA) was added twice, with a vortex mix for 30 s after each addition. Subsequently, 400 µl of chloroform and 400 µl of NaHCO_3_ solution were sequentially added, with a vortex mix for 10 s after each addition. To facilitate phase separation, the blended liquid was centrifuged at 2000 rpm for 10 min, and the upper water and intermediate protein layers were carefully discarded. Following this step, sodium sulfate, similar to mung beans, was added to the lowest chloroform layer for water absorption. Finally, 200 µl of the resulting liquid phase, containing derivatized metabolites, was transferred into the GS automatic sampler sample bottle for subsequent analysis.

### 2.5 Instrumentation and parameters for metabolomic profiling

This study utilized an Agilent Intuvo 9000 gas chromatograph coupled to an Agilent MSD5977B mass spectrometer detector employing electron-impact ionization(70 eV) to analyze derivatized metabolites. Metabolite separation was achieved using a BD-1701 gas phase capillary column (30 m × 0.25 mm × 0.25 μm film thickness, Agilent Technologies).

The GC inlet was configured in splitless mode, with the injector temperature set at 290 °C. Helium was the carrier gas at a flow rate of 1.0 ml/min. The GC oven temperature followed a gradient protocol, starting at 45 °C and increasing to 180 °C at a rate of 9.0 °C/min, then to 220 °C at 40.0 °C/min, followed by an increase to 240 °C at 40.0 °C/min, and finally reaching 280 °C at 80.0 °C/min. The temperature settings for the auxiliary, MS quadrupole, MS source, and guard chip were 250 °C, 230 °C, 150 °C, and 280 °C, respectively. Mass detection occurred within the range of 50 μm to 550 μm, with a scan speed of 1.562 µs after a solvent delay of 5.5 min.

Metabolite deconvolution was facilitated by the Automatic Mass Spectrometry Deconvolution & Identification system software. Metabolite identification relied on comparing MS fragmentation patterns, specifically the mass-to-charge ratio and relative strength of the mass spectrum, alongside respective GC retention times, referencing an in-house MS library constructed with chemical standards. Any remaining putative compounds were identified using a commercial NIST mass spectral library. Relative concentrations of metabolites were extracted using a MassOmics R-based script through the peak height of the most abundant fragment ion mass.

###  Absolute quantification of metabolite concentration

Each metabolite analyzed was paired with a corresponding chemical standard for quantification. Chemical compound standards, including typical amino acids, fatty acids, and glucose metabolites, were utilized for this purpose. A standard curve for these metabolites was established based on the peak height corresponding to their concentrations. Subsequently, the concentration of metabolites detected in serum samples was normalized by the internal standard and quantified according to the standard curve established previously.

###  Quality control

Quality Control (QC) samples were incorporated into this study to ensure data quality and mitigate batch-to-batch variations. Preparing QC samples involved mixing samples of the same volume to be tested and subjecting them to the same pre-treatment method as the samples under investigation. Subsequently, the processed QC samples were introduced into the GC-MS system alongside the test samples for detection and analysis. The operation followed the protocol of inserting a processed 20 µl QC sample after every 15 test samples. Each metabolite’s relative standard deviation (RSD) was computed during the data processing stage. Metabolites with an RSD > 30% were excluded from further data analysis.

###  Data processing and statistical analysis

The Human Metabolome Database (HMDB) and PubChem were referenced to retrieve and further identify the detected metabolites in this study. Subsequently, all metabolite concentration values underwent normalization through Metaboanalyst 5.0 (www.metaboanalyst.ca). The differential metabolites were identified through a combined approach utilizing the False Discovery Rate (FDR) from the t-test and the Variable Importance in Projection (VIP) value based on the Orthogonal Partial Least Squares Discriminant Analysis (OPLS-DA) model. The threshold criteria were VIP > 1.0, *P* value < 0.05, and FDR < 0.2. The Kyoto Encyclopedia of Genes and Genomes (KEGG) analysis was conducted to elucidate the potential pathways involved. Binary logistic regression models, adjusted for age, BMI, and AMH as covariates, were utilized to estimate odds ratios (ORs) and 95% confidence intervals (CIs) for the relationship between differential metabolites and the risk of HOR. Metabolites with significant associations (*P* value < 0.05) were identified as related to HOR risk within the defined groups. Spearman correlation analysis examined the associations between metabolites and clinical characteristics. Correlations with coefficients (R2) ≥ 0.2 or R2 ≤ -0.2 and *P* value < 0.05 were deemed statistically significant. All statistical analyses and visualizations were executed using the R software.

##  Results

###  Clinical characteristics of participants

In this study, 145 infertile patients were categorized into two main groups: 80 in the CON group and 65 in the PCOS group. Within the CON group, there were two subgroups: CON-NOR (*n* = 40) and CON-HOR(*n* = 40). Similarly, within the PCOS group, there were two subgroups: PCOS-NOR (*n* = 26) and PCOS-HOR (*n* = 39).

**Fig. 1:**
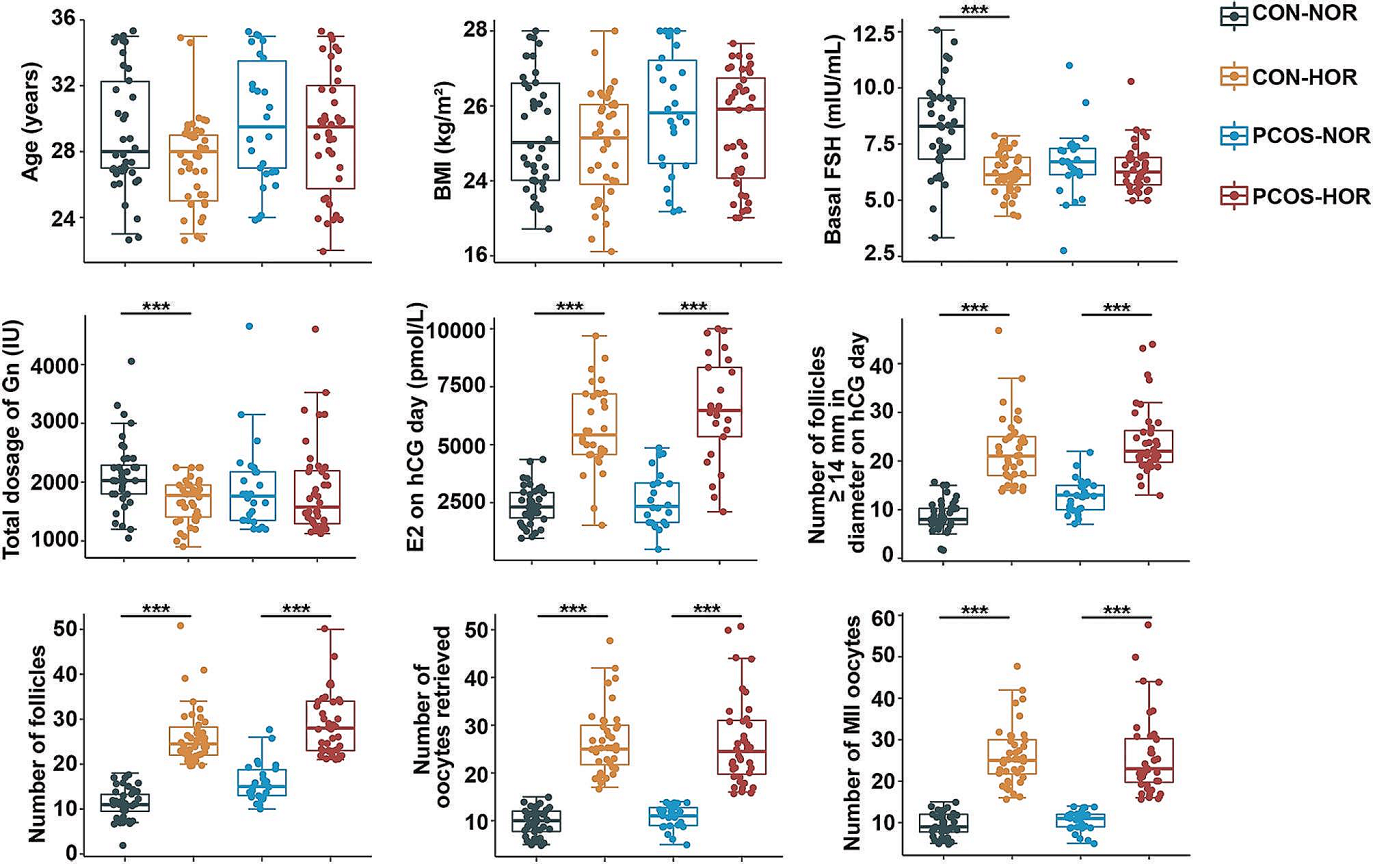
Comparative Analysis of Clinical Characteristics Comparative analysis of clinical characteristics among different subgroups was conducted using the Wilcoxon rank-sum test. The legend indicates key abbreviations: CON (Tubal infertility), PCOS (Polycystic Ovarian Syndrome), NOR (Normal Ovarian Response), HOR (High Ovarian Response and at risk for OHSS), BMI (Body Mass Index), FSH (Follicle-Stimulating Hormone), E2 (Estradiol), Gn (Gonadotropin), and hCG (Human Chorionic Gonadotropin). Statistical significance was set at *P* < 0.05 (* *P* < 0.05; ** *P* < 0.01; *** *P* < 0.001)

The clinical characteristics are depicted in Fig. 1A and Supplementary Table 1. Notably, within the CON group, the CON-HOR subgroup displayed lower basal serum FSH levels than the CON-NOR subgroup. However, no such difference was observed between the two PCOS subgroups. Furthermore, no significant differences were found between NOR and HOR patients in both CON and PCOS groups regarding other characteristics such as age, BMI, basal serum E2 levels and basal P levels. Additionally, HOR patients in both groups demonstrated significantly higher levels of various characteristics, including E2 level on hCG day, the number of follicles ≥ 14 mm and ≥ 17 mm in diameter on hCG day, as well as the number of follicles, oocytes retrieved, mature oocytes, and MII oocytes compared to the NOR patients.

###  Metabolic landscape within subgroups

A total of 125 different metabolites were identified in this study. Principal Component Analysis (PCA) initially indicated no significant difference in metabolic profiles between NOR and HOR patients in CON and PCOS groups (data not presented). To further verify the differences between NOR and HOR patients, we proceeded with OPLS-DA analysis to maximize the differences between subgroups in the model. The results demonstrated a significant divergence between NOR and HOR patients within the CON and PCOS groups (Supplementary Fig. 2).

**Fig. 2:**
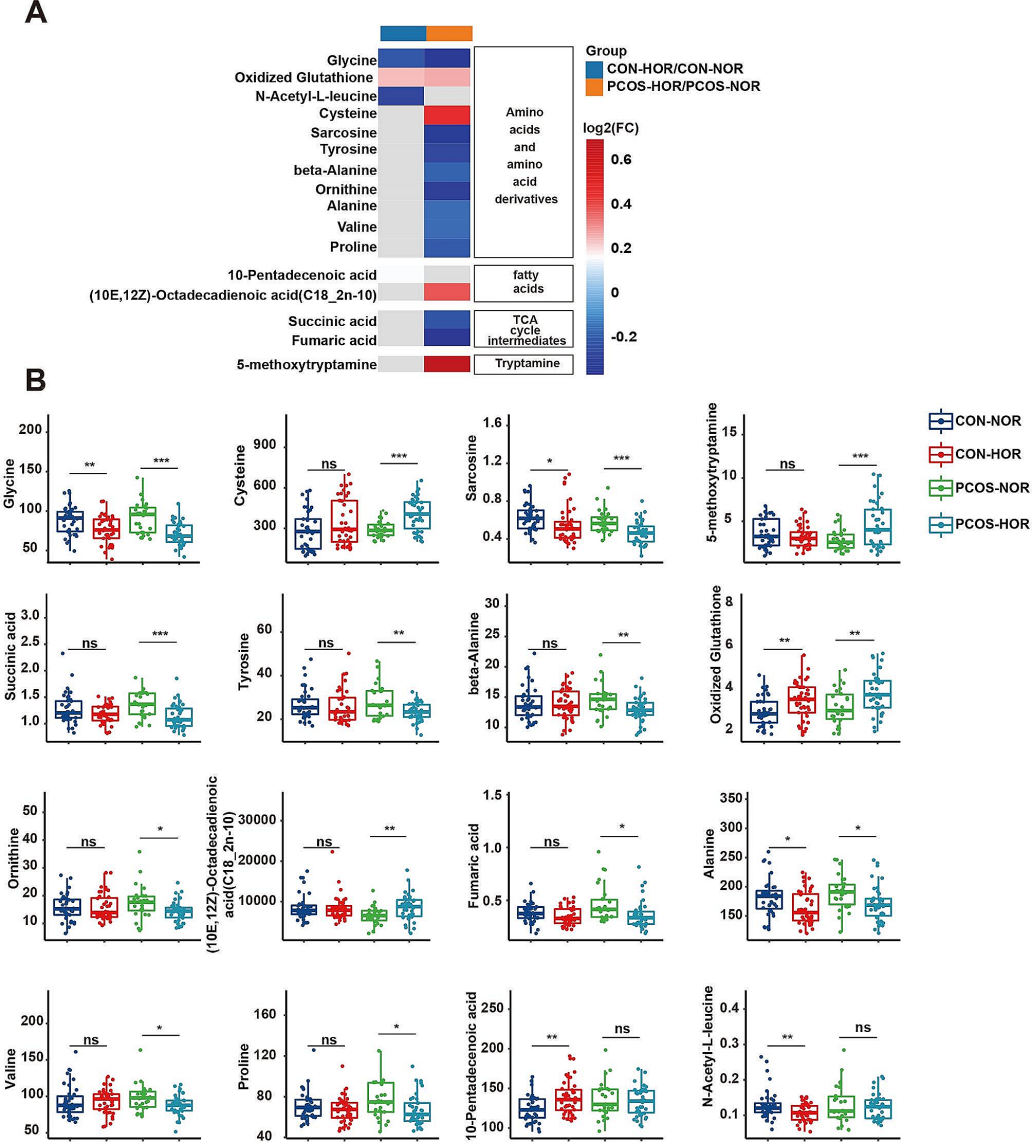
Differential metabolic profiles in serum of HOR patients (**A**) Heatmap illustrating the detected differential metabolites across each group, indicating the ratio of metabolite levels in subgroup comparisons. The color gradient reflects concentration disparities, with red indicating higher concentrations in the HOR group compared to the NOR group and blue representing lower concentrations. (**B**) The box and scatter plots display the concentrations of differential metabolites across various subgroups. Statistical significance was set at *P* < 0.05 (* *P* < 0.05; ** *P* < 0.01; *** *P* < 0.001), while ‘ns’ denotes non-significance

In the CON group, four metabolites exhibited significant differences between the CON-NOR and CON-HOR subgroups (*P* < 0.05, FDR < 0.2, VIP > 1): 10-pentadecenoic acid, glycine oxidized, glutathione, and N-acetyl-L-leucine (Fig. 2A and Supplementary Table 2). Similarly, in the PCOS group, 14 metabolites serum metabolites displayed significant variances between the PCOS-NOR and PCOS-HOR subgroups (*P* < 0.05, FDR < 0.2, VIP > 1). Among these metabolites, eight were amino acid metabolites, including glycine, N-acetyl-L-leucine, sarcosine, tyrosine, beta-alanine, ornithine, alanine, and valine, with lower levels observed in the PCOS-HOR subgroup (Fig. 2 and Supplementary Table 3). Furthermore, oxidized glutathione, another amino acid metabolite, was elevated in the PCOS-HOR subgroup. Additionally, tricarboxylic acid cycle (TCA) metabolites, such as succinic acid and fumaric acid, showed significantly lower levels in the PCOS-HOR subgroup compared to the PCOS-NOR subgroup. Similarly, fatty acid metabolites such as (10E,12Z)-octadecadienoic acid (C18_2n-10) and tryptamine metabolites like 5-methoxytryptamine were notably elevated in the PCOS- HOR subgroup compared to the PCOS-NOR subgroup.

Interestingly, glycine and oxidized glutathione emerged as differentiated metabolites between NOR and HOR patients in both the CON and PCOS groups. Specifically, glycine levels consistently trended lower in HOR patients compared to NOR patients across both groups, while oxidized glutathione levels consistently trended higher in HOR patients (Fig. 2).

###  KEGG enrichment analysis of key metabolic signatures in all participants

**Fig. 3:**
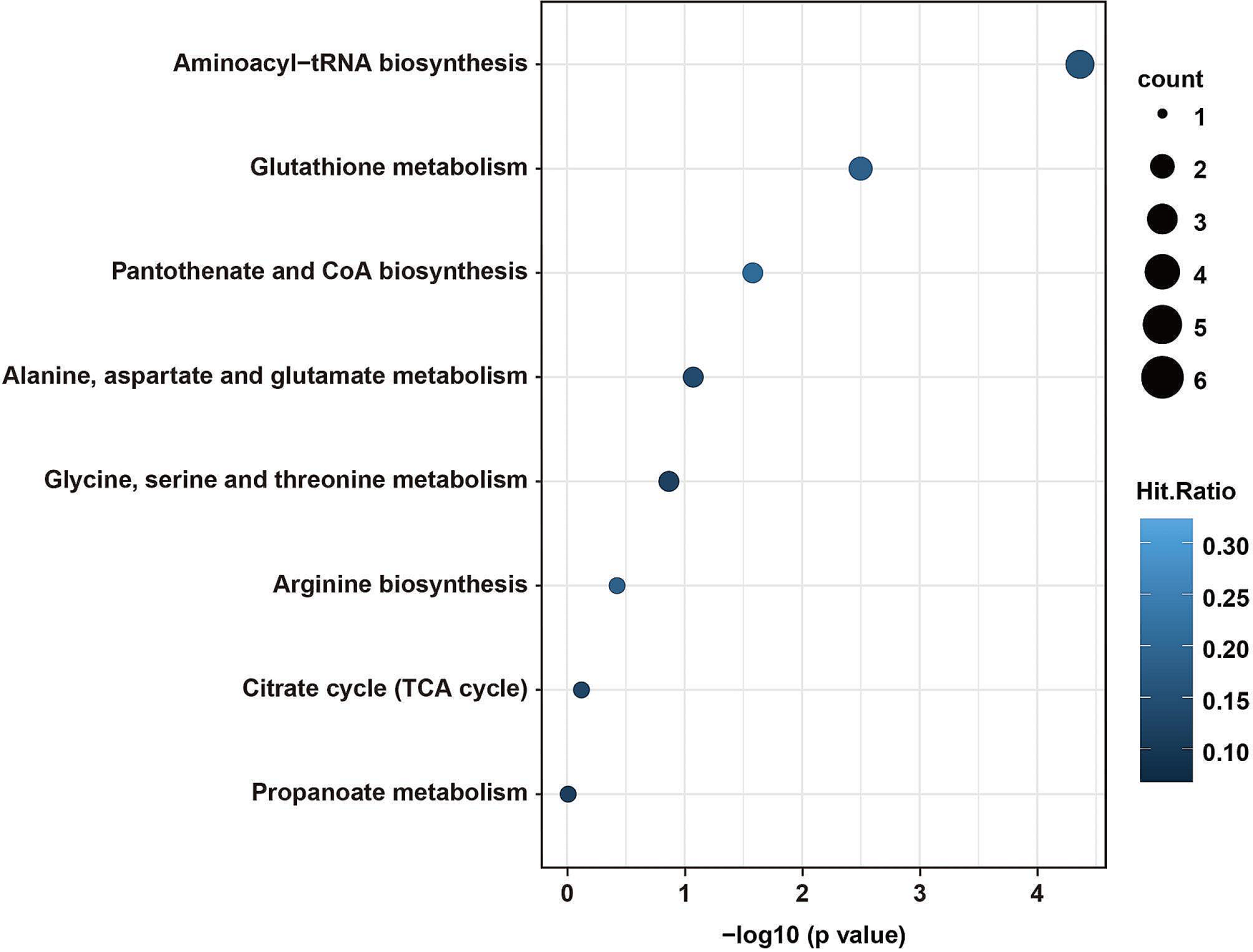
KEGG pathway enrichment analysis of differential metabolites in PCOS subgroup KEGG pathway enrichment analysis of differential metabolites identified in the comparison between the PCOS-NOR and PCOS-HOR subgroups. The vertical axis represents distinct metabolic pathways, while the horizontal axis indicates the Holm-adjusted *P* value. The circle size reflects the number of Hits, and the color indicates Hits.Ratio

We conducted a KEGG enrichment analysis on the identified differential metabolites. In the CON group, where only four metabolites displayed significant differences between the CON-NOR and CON-HOR subgroups, the decision was made to discontinue the KEGG analysis for this group. However, for the PCOS-NOR and PCOS-HOR subgroups, encompassing 14 differential metabolites, KEGG analysis revealed significant enrichment across several pivotal pathways, namely aminoacyl-tRNA biosynthesis, glutathione metabolism, and pantothenate and CoA biosynthesis (Fig. 3). These pathways are crucial for maintaining essential cellular processes and physiological functions. Specifically, aminoacyl-tRNA biosynthesis is vital for protein synthesis [21], glutathione metabolism plays a key role in cellular defense against oxidative stress [22], and pantothenate and CoA biosynthesis is critical for energy metabolism and the TCA cycle [23].

###  Identification of HOR Risk-Associated metabolites

**Fig. 4:**
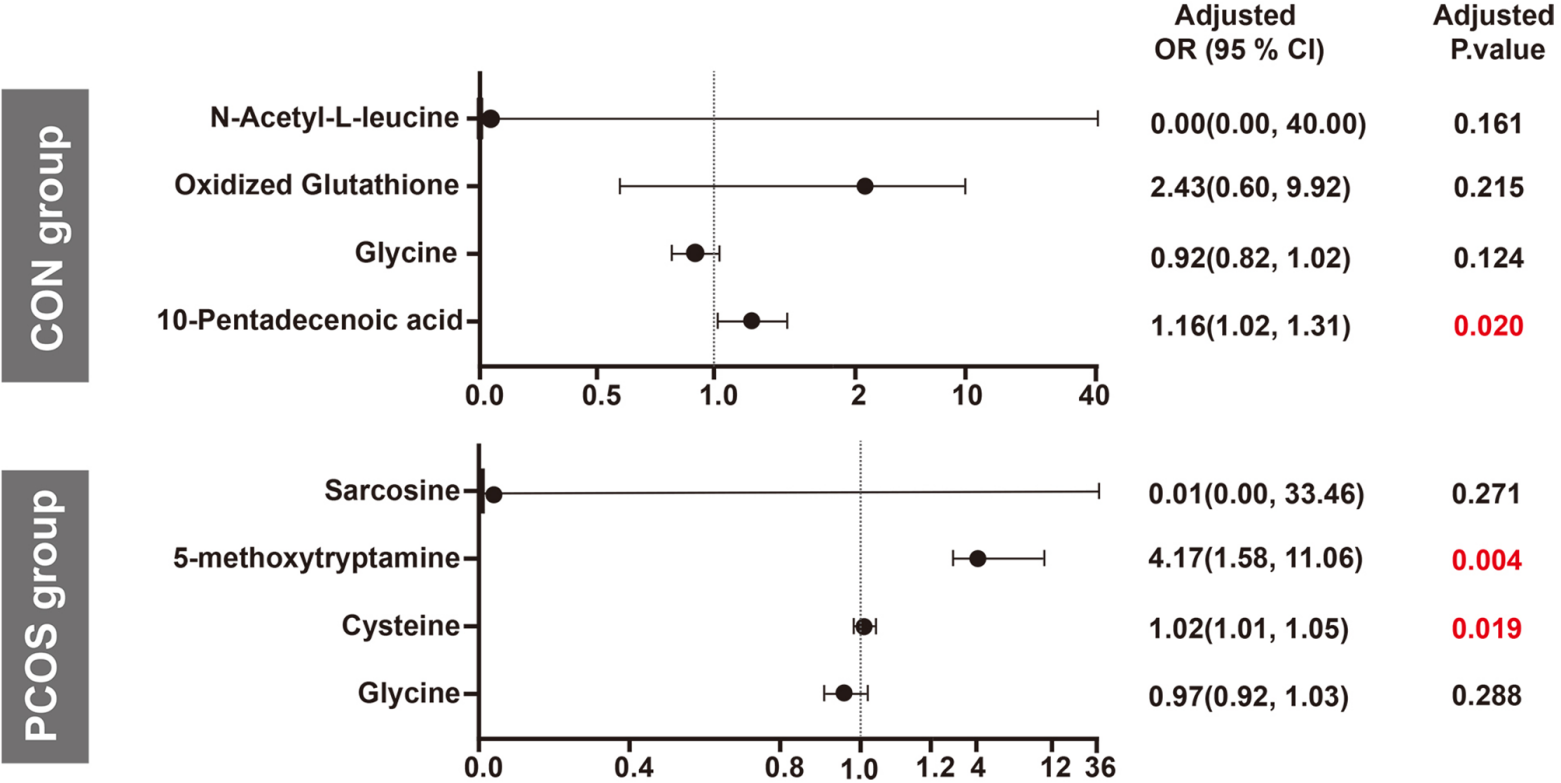
Associations between differential metabolites for HOR: adjusted OR (95% CIs) from binary logistic regression analysis aOR: adjusted odds ratio; CI: confidence interval

After thorough adjustments for age, BMI, and AMH using binary logistic regression, a specific differential metabolite emerged as significantly associated with CON-HOR risk in the CON group. Specifically, 10-pentadecenoic acid demonstrated an increased risk of CON-HOR by 1.16-fold (95% CI 1.02–1.31; *P*=0.020) with each standard deviation (SD) increment (Fig. 4 and Supplemental Table 4).

In the PCOS group, we observed 14 differential metabolites between the PCOS-NOR and PCOS-HOR subgroups. Due to the limitations in sample size, we prioritized metabolites for further analysis based on the VIP scores. Specifically, we focused on the top four metabolites (*P* < 0.001, FDR < 0.1) for subsequent binary logistic regression analysis. Notably, after adjustments for age, BMI, and AMH, our findings revealed that the risk of PCOS-HOR escalated by 1.02 (95% CI 1.01–1.05; *P*=0.019) with each SD increase in cysteine levels and 4.17 (95% CI 1.58–11.16; *P*=0.004) with each SD increase in 5-methoxytryptamine levels (Fig. 4 and Supplemental Table 5).

###  Correlations of Differential Metabolite with Clinical Characteristics in NOR and HOR Subgroups of CON and PCOS

**Fig. 5:**
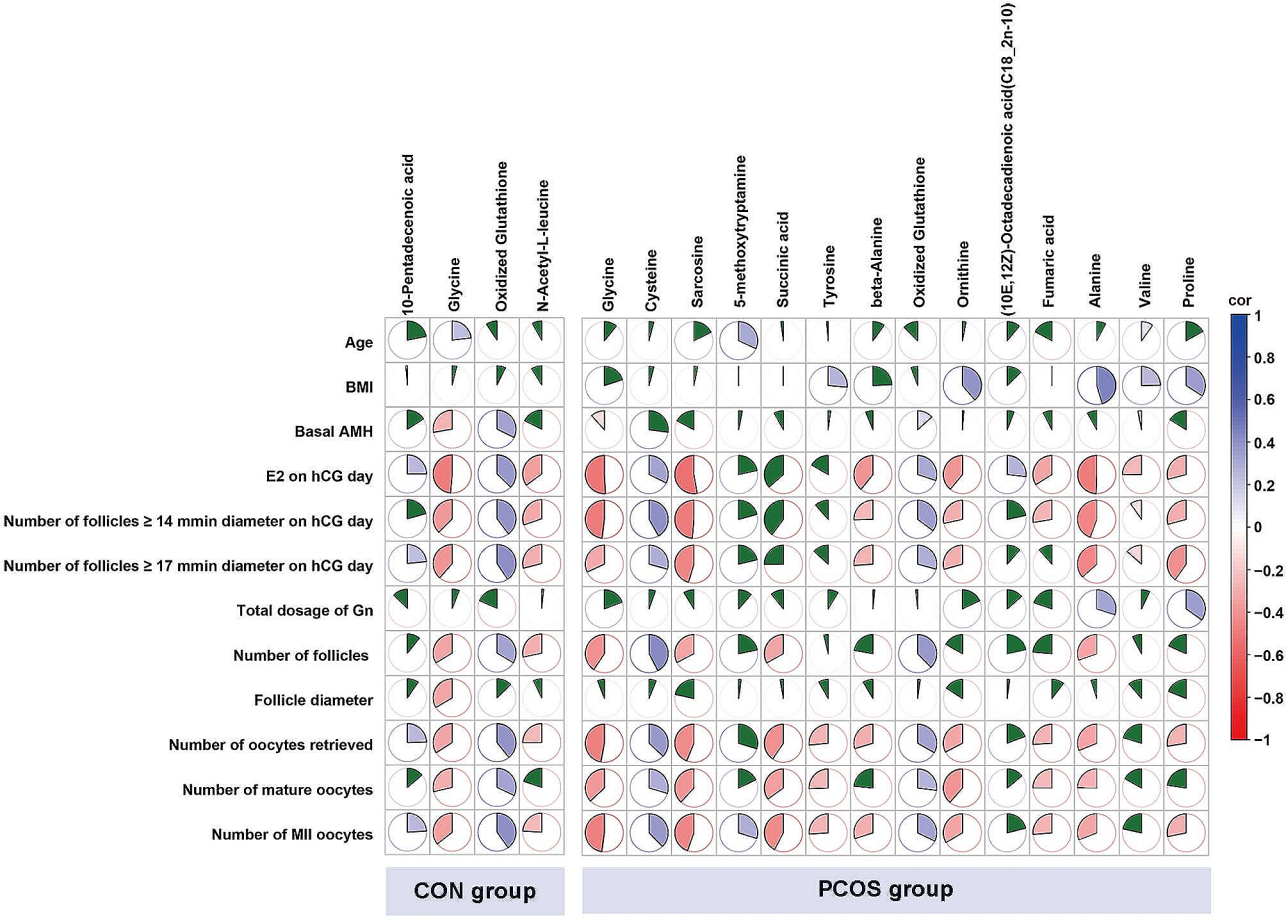
Spearman correlation analysis between differential metabolites and clinical characteristics Positive correlations are denoted in blue, while negative correlations are depicted in red. The size of each sector corresponds to the correlation coefficient. The green-filled sectors represent correlations lacking statistical significance

Four differential metabolites differed between the CON-NOR and CON-HOR subgroups in the CON group. These four metabolites showed significant correlations with the number of oocytes retrieved and MII oocytes. However, none correlated with the total dose of Gn (Fig. 5 and Supplemental Tables 6–7).

Within the PCOS group, 14 metabolites differed significantly between the PCOS-NOR and PCOS-HOR subgroups. Among these, excluding (10E,12Z)-octadecadienoic acid (C18_2n-10) and valine, the remaining 12 metabolites demonstrated notable correlations with the number of oocytes retrieved and MII oocytes. Noteworthy is that only alanine and proline displayed significant correlations with the total dosage of Gn, while the others showed no such correlation (Fig. 5 and Supplemental Tables 8–9).

Interestingly, glycine and oxidized glutathione emerged as differential metabolites between NOR and HOR patients in both the CON and PCOS groups. In both groups, glycin displayed significant negative correlations with basal AMH levels, E2 levels on hCG day, the number of follicles ≥ 14 mm and ≥ 17 mm in diameter on hCG Day, as well as the number of oocytes retrieved, mature oocytes, and MII oocytes. Conversely, oxidized glutathione demonstrated significant positive correlations with these clinical characteristics (Fig. 5 and Supplemental Tables 6–9).

### 3.6. Unveiling Shared metabolic signatures: insights into PCOS and HOR Pathogenesis

**Fig. 6:**
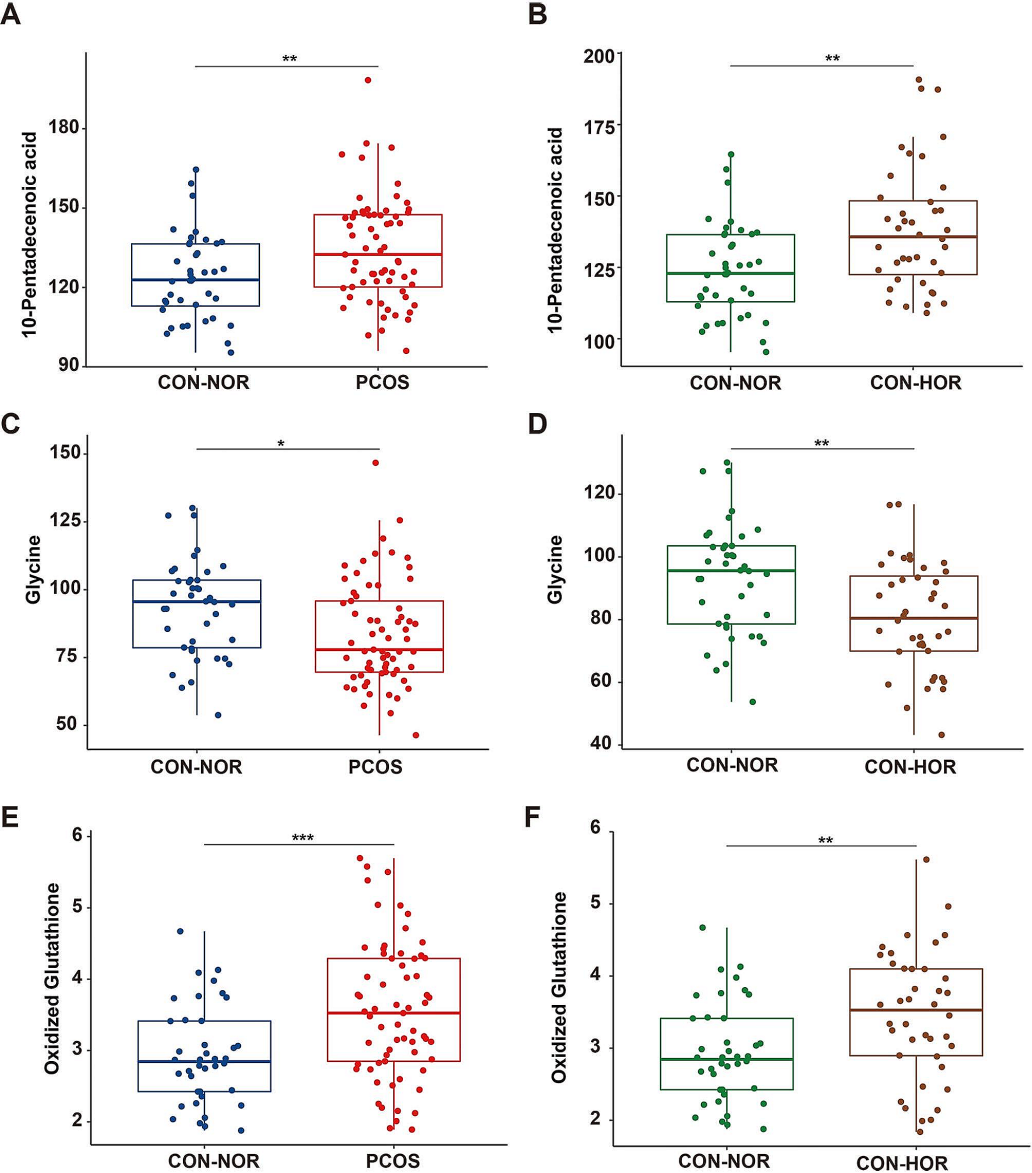
Comparative metabolite profiling in PCOS and CON subgroups The box plot and scatter plot illustrate the concentrations of differential metabolite between PCOS and CON subgroups. Panels (**A**, **C**, **E**) display the metabolite concentrations between the CON-NOR and PCOS groups, while panels (**B**, **D**, **F**) show the metabolite concentrations between the CON-NOR and CON-HOR subgroups

To investigate potential similarities in metabolic profiles between PCOS and HOR, we analyzed metabolite changes within the PCOS group. Our findings revealed 29 metabolites identified as differential between the PCOS and CON-NOR groups (*P* < 0.05, FDR < 0.2, VIP > 1, Supplementary Table 10). Notably, three of these metabolites—10-pentadecenoic acid, glycine, and oxidized glutathione—were also identified as differentially expressed between the CON-NOR and CON-HOR groups (Supplemental Fig. 3A). Further analysis demonstrated that these three metabolites exhibited significant upregulation in both the PCOS and CON-HOR groups relative to the CON-NOR group (Fig. 6). This consistent alteration suggests a parallel in metabolic profiles between PCOS and HOR, hinting at a potential shared etiological similarity between the two conditions.

Furthermore, KEGG pathway analysis of these metabolites revealed significant enrichment in aminoacyl-tRNA biosynthesis and glutathione metabolism pathways (Supplementary Fig. 3B). These findings support earlier KEGG pathway analysis of differential metabolites in PCOS subgroups, indicating a close link between these pathways and the development of PCOS and PCOS-HOR.

##  Discussion

In this study, we utilized GC-MS to investigate the serum metabolic profiles of patients with normal ovarian response and those exhibiting high ovarian response and at risk for OHSS in tubal and PCOS infertility. Our analysis revealed the identification of 4 and 14 differential metabolites between these two ovarian response patient subsets in each infertility background, respectively. Additionally, we observed a certain degree of similarity in the metabolic profiles between PCOS patients and those exhibiting high ovarian response and at risk for OHSS.

Previous studies have highlighted significant differences in amino acid and lipid metabolites within the follicular fluid of OHSS patients compared to control groups [13, 17, 24]. Similarly, multiple lipid components in the serum or follicular fluid of PCOS patients exhibit alterations [25, 26], particularly noticeable in obese individuals with PCOS, where lipid abnormalities are most pronounced [27]. In line with these findings, our study also revealed abnormal elevations in several lipid metabolites, including myristic acid (C14_0), 10-pentadecenoic acid, lignoceric acid (C24_0), nervonic acid (C24_1n-9c), and hexanoic acid (C6_0), within the serum of PCOS group when compared to CON-NOR group.

Furthermore, some researchers explored the metabolic profiles of populations exhibiting different ovarian responses. Mu et al. [28] discovered that elevated glycine levels are associated with a heightened response to COS. Interestingly, our study contradicts this finding, as we observed lower glycine levels in the HOR patients across both infertility backgrounds. Additionally, glycine also showed negative correlations with several clinical characteristics, including levels of AMH, the number of oocytes retrieved, mature oocytes, and MII oocytes. The propensity for ovarian hyperstimulation significantly escalates in PCOS patients following COS stimulation [15, 29]. Several metabolomic studies on PCOS patients have consistently documented significantly reduced glycine levels compared to controls [30–32], which aligns with our results of lower glycine levels in the PCOS group compared to the CON-NOR group. Furthermore, research has indicated heightened glycine expression in women with diminished ovarian reserve (DOR) [33], which is correlated with a poor response to ovarian stimulation [34]. However, it is noteworthy that a research has found a positive correlation between glycine and one marker of ovarian reserve, the antral follicle count (AFC) [35]. Considering these diverse research findings, the role of glycine in ovarian response remains unclear. Future studies should incorporate larger sample sizes from various regions and employ more rigorous experimental designs to elucidate the relationship between glycine and ovarian response.

Hood et al. [14] discovered that most fatty acids and amino acids in the serum metabolome correlate with the number of mature oocytes. Our study echoes these findings, as most differential metabolites between NOR and HOR patients are within the amino acid class. Moreover, these differential metabolites, including alanine, cysteine, glycine, ornithine, glutathione oxide, sarcosine, and tyrosine, demonstrate significant associations with the number of mature oocytes. This alignment underscores the importance of amino acid metabolism in modulating ovarian response and highlights its potential as a target for interventions to optimize oocyte yield.

Furthermore, our study revealed a significant enrichment of differential metabolites in the aminoacyl-tRNA biosynthesis pathway, both between PCOS-NOR and PCOS-HOR groups, and between PCOS and CON-NOR groups. This finding aligns with the investigation by Li et al., who focused on metabolic profiles in patients with DOR [36]. In their study, researchers noted a similar trend, wherein the metabolites distinguishing the DOR patients from patients with normal ovarian reserve were prominently associated with the aminoacyl-tRNA biosynthesis pathway [36]. The aminoacyl-tRNA biosynthesis pathway plays a pivotal role in cellular processes by coupling amino acids with their corresponding tRNA molecules, forming aminoacyl-tRNA complexes crucial for protein synthesis [21, 37]. This fundamental process ensures the precise integration of amino acids into nascent polypeptide chains, thereby dictating proteins’ ultimate structure and function. These findings imply a potential link between the aminoacyl-tRNA biosynthesis pathway and reproductive disorders like PCOS and DOR. Nevertheless, further investigation is required to ascertain whether this pathway influences the onset of these conditions through its impact on protein synthesis.

In summary, this study identified the serum metabolic profiles of patients exhibiting high ovarian response and at risk for OHSS. However, several limitations should be acknowledged. Firstly, the relatively small overall sample size and the sole reliance on samples from a single medical institution limit the generalizability of our findings. Furthermore, this constraint impedes randomization and stratification of the sample, necessitating caution in interpreting our results. Secondly, to mitigate potential confounding effects, we applied stringent inclusion criteria for participant recruitment, restricting the age range to 20–35 years and setting a BMI threshold of ≤ 28 kg/m² for both groups. Hence, our study did not include older individuals or those with a higher BMI, and therefore, our findings may not fully represent the metabolic profile of the entire PCOS patients. Thirdly, our results offer an overview of metabolic aspects in PCOS patients but lack the detailed information required to identify different phenotypes within the patients. As a result, we are unable to characterize the unique metabolic profiles associated with these phenotypes.

## Conclusions

Utilizing GC-MS technology, we described the metabolic profiles of specific patients who exhibit high ovarian response and are at risk for OHSS within both the tubal infertility group and the PCOS infertility group. The discovery of risk metabolites in these unique patients provides clues to studying their pathological mechanisms. Further, similarities in metabolic profiles between patients with PCOS and high ovarian response suggest a potential common underlying cause, urging more research into their connection.

### Electronic supplementary material

Below is the link to the electronic supplementary material.

Supplementary Material 1

## Data Availability

To access the data supporting the findings of this study, kindly contact the corresponding author. They will be pleased to provide the necessary information upon request.

## Abbreviations

OHSS: Ovarian hyperstimulation syndrome
CON: Tubal factor infertility
PCOS: Polycystic ovary syndrome
NOR: Normal ovarian response
HOR: High ovarian response and at risk for OHSS
GC-MS: Gas chromatography-mass spectrometry
IVF: In vitro fertilization
KEGG: Kyoto Encyclopedia of Genes and Genomes
