# Supplementary material for: Metabolic landscape and pathogenic insights: a comprehensive analysis of high ovarian response in infertile women undergoing in vitro fertilization
Source: J Ovarian Res. 2024 May 17;17:105. doi: 10.1186/s13048-024-01411-6 (PMC11102248; doi:10.1186/s13048-024-01411-6)
Supplement: Supplementary file 1 — Supplementary Material 1 [file 13048_2024_1411_MOESM1_ESM.docx]

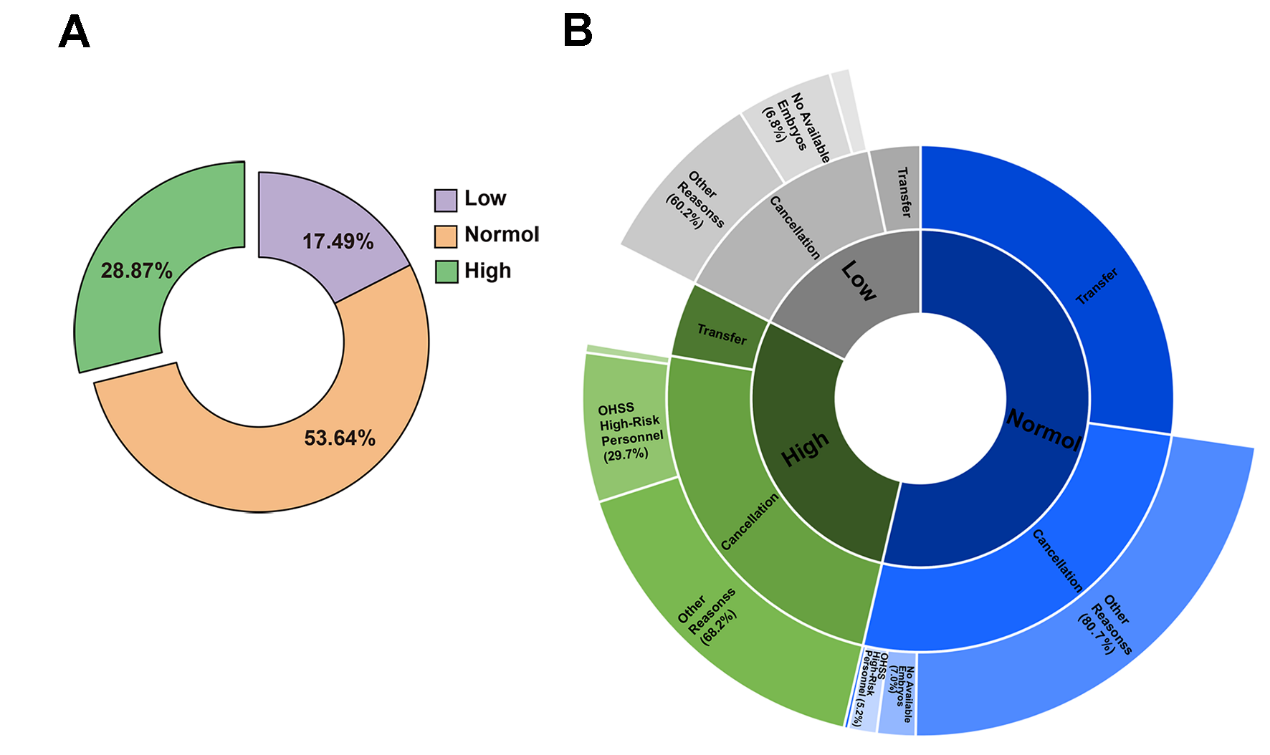


# Supplementary Fig 1. Distribution of Ovarian Responses

# Supplementary Table 1. Clinical Characteristics of Participants in the CON and PCOS Groups.

|  | **CON-NOR**  **(n = 40)** | **CON-HOR**  **(n = 40)** | ***P* value^a^** | **PCOS-NOR**  **PC (n = 26)** | **PCOS-HOR**  **PC (n =39)** | ***P* value^b.^** |
| --- | --- | --- | --- | --- | --- | --- |
| Age, median (IQR), (years) | 28.00 (27.00 - 35.00) | 28.00 (25.00 - 29.00) | 0.082 | 29.50 (27.00 - 33.50) | 29.50 (25.75 - 32.00) | 0.464 |
| BMI, median (IQR), (kg/m^2)^ | 22.05 (20.03 - 25.21) | 22.30 (19.82 - 24.07) | 0.277 | 23.63 (20.93 - 26.43) | 23.83 (20.15 - 25.49) | 0.245 |
| Basal FSH, median (IQR), (mIU/mL) | 8.29 (6.83 - 9.54) | 6.13 (5.69 - 6.91) | **<.001** | 6.71 (6.14 - 7.30) | 6.25 (5.69 - 6.91) | 0.194 |
| Basal E2, median (IQR), (pmol/L) | 33.50 (28.00 - 38.00) | 34.50 (28.00 - 41.00) | 0.528 | 31.50 (25.25 - 41.00) | 33.00 (28.00 - 39.50) | 0.541 |
| Basal P, median (IQR), (ng/mL） | 0.57 (0.36 - 0.76) | 0.57 (0.39 - 0.80) | 0.899 | 0.43 (0.34 - 0.65) | 0.53 (0.28 - 0.79) | 0.753 |
| Basal LH, median (IQR), (mIU/mL) | 3.31 (2.58 - 4.16) | 4.03 (3.15 - 5.67) | **0.030** | 4.03 (2.44 - 11.32) | 5.85 (4.68 - 8.67) | 0.111 |
| Basal AMH, median (IQR), (ng/mL) | 2.24 (1.60 - 4.15) | 7.19 (5.54 - 8.71) | **<.001** | 6.62 (5.20 - 9.76) | 10.72 (7.10 - 13.64) | **0.002** |
| FSH on initial Gn day, median (IQR), (mIU/mL) | 8.04 (6.83 - 9.54) | 6.13 (5.69 - 6.90) | **<.001** | 6.70 (6.14 - 7.30) | 6.25 (5.69 - 6.91) | 0.225 |
| E2 on initial Gn day, median (IQR), (pmol/L) | 33.00 (27.75 - 38.50) | 35.50 (28.00 - 40.25) | 0.470 | 32.00 (25.25 - 46.00) | 34.50 (28.00 - 42.25) | 0.568 |
| LH on initial Gn day, median (IQR), (mIU/mL) | 3.45 (2.64 - 4.20) | 4.12 (3.15 - 5.83) | **0.039** | 3.93 (2.21 - 9.12) | 6.63 (4.66 - 10.25) | **0.041** |
| P on initial Gn day, median (IQR), (nmoL/L） | 0.57 (0.35 - 0.73) | 0.59 (0.40 - 0.74) | 0.848 | 0.51 (0.35 - 0.66) | 0.51 (0.28 - 0.89) | 0.989 |
| FSH on hCG day, median (IQR), (mIU/mL) | 14.62 (11.64 - 17.17) | 11.97 (10.60 - 14.16) | **0.017** | 12.36 (11.07 - 14.70) | 10.61 (8.36 - 12.63) | **0.040** |
| E2 on hCG day, median (IQR), (pmol/L) | 2312.50 (1842.00 - 2933.25) | 5421.50 (4575.25 - 7189.25) | **<.001** | 2340.00 (1652.50 - 3346.75) | 6477.00 (5345.00 - 8331.00) | **<.001** |
| LH on hCG day, median (IQR), (mIU/mL) | 2.15 (1.30 - 3.23) | 1.56 (1.08 - 2.78) | 0.199 | 2.59 (1.68 - 5.27) | 2.26 (1.26 - 4.17) | 0.272 |
| P on hCG day, median (IQR), (nmoL/L） | 0.79 (0.45 - 1.10) | 1.29 (1.01 - 1.95) | **<.001** | 0.75 (0.55 - 1.17) | 1.31 (0.89 - 1.89) | **0.003** |
| Number of follicles ≥ 14 mm in diameter on hCG day, median (IQR) | 8 (7 - 10) | 21 (17 - 25) | **<.001** | 13 (10 - 15) | 22 (20 – 27) | **<.001** |
| Number of follicles ≥ 17mm in diameter on hCG day, median (IQR) | 5 (5 - 7) | 13 (11 - 14) | **<.001** | 7 (6 - 9) | 13 (10 – 16) | **<.001** |
| Endometrial thickness on hCG day, median (IQR), (mm) | 10.50 (9.00 - 12.00) | 10.75 (9.88 - 13.00) | 0.533 | 11.00 (9.00 - 12.50) | 10.00 (9.25 - 11.00) | 0.183 |
| Initial dosage of Gn, median (IQR), (IU) | 225.00 (225.00, 225.00) | 200.00 (150.00, 225.00) | **<.001** | 200.00 (150.00, 225.00) | 150.00 (150.00, 225.00) | 0.215 |
| Total duration of GN, median (IQR), (days) | 9 (9, 10) | 9 (9, 9) | 0.720 | 9 (8, 10) | 9 (9, 10) | 0.302 |
| GN type, No.(%) |  | | | | | |
| Urofollitropin | 5 (12.50) | 6 (15.00) | 0.139 | 3 (11.54) | 4 (10.00) | 0.398 |
| Menotropins | 7 (17.50) | 0 (0.00) |  | 2 (7.69) | 1 (2.50) |  |
| Puregon | 10 (25.00) | 12 (30.00) |  | 7 (26.92) | 10 (25.00) |  |
| Gonal-F | 5 (12.50) | 7 (17.50) |  | 2 (7.69) | 10 (25.00) |  |
| Fostimon | 1 (2.50) | 3 (7.50) |  | 1 (3.85) | 0 (0.00) |  |
| Menopur | 1 (2.50) | 1 (2.50) |  | 3 (11.54) | 2 (5.00) |  |
| Kim Sai Heng | 11 (27.50) | 11 (27.50) |  | 8 (30.77) | 13 (32.50) |  |
| Total dosage of Gn, median (IQR), (IU) | 2025.00 (1800.00 - 2287.50) | 1775.00 (1406.25 - 1950.00) | **<.001** | 1762.50 (1350.00 - 2175.00) | 1575.00 (1293.75 - 2193.75) | 0.645 |
| Duration of infertility, median (IQR), (years) | 2.50 (1.75 - 3.25) | 2.00 (1.75 - 4.00) | 0.965 | 4.00 (2.25 - 5.00) | 3.00 (2.00 - 5.00) | 0.414 |
| Number of follicles, median (IQR) | 11 (10 - 13) | 24 (21 - 28) | **<.001** | 16 (13 - 19) | 28 (23 - 34) | **<.001** |
| Mean follicle diameter, median (IQR), (mm) | 15.52 (14.88 - 16.32) | 16.46 (16.00 - 16.97) | **0.003** | 15.96 (15.10 - 16.83) | 16.29 (15.29 - 16.84) | 0.670 |
| Number of oocytes retrieved, median (IQR) | 10 (8 - 12) | 25 (22 - 30) | **<.001** | 11 (9 - 13) | 25 (20 - 31) | **<.001** |
| Number of mature oocytes, median (IQR) | 8 (6 - 10) | 22 (18 - 28) | **<.001** | 10 (5 - 12) | 21 (16 - 26) | **<.001** |
| Number of MII oocytes, median (IQR) | 9 8 - 12) | 25 (22 - 30) | **<.001** | 11 (9 - 12) | 23 (20 - 30) | **<.001** |

^a.^ *P* value for CON-NOR and CON-HOR comparison.

^b.^ *P* value for PCOS-NOR and PCOS-HOR comparison.

The bold fonts mean the *P* value < 0.05.

BMI=body mass index, AMH=anti-Müllerian hormone, P= progesterone, FSH=follicle-stimulating hormone, LH=luteinizing hormone, E2=estradiol, Gn= gonadotropin, hCG=human Chorionic Gonadotropin.

# Supplementary Table 2. Basic Information on Differential Metabolites between CON-NOR and CON-HOR

| **Metabolite** | **Type** | ***P* value** | **VIP** | **log2(FC)** | **FDR** |
| --- | --- | --- | --- | --- | --- |
| 10-Pentadecenoic acid | FA | <.001 | 2.60 | 0.16 | 0.12 |
| Glycine | AA | <.001 | 2.43 | -0.21 | 0.12 |
| Oxidized Glutathione | AA | <.001 | 2.06 | 0.25 | 0.12 |
| N-Acetyl-L-leucine | AA | <.001 | 2.76 | -0.28 | 0.14 |

Note: FC= Fold Change, VIP= Variable Importance in Projection value based on the Orthogonal Partial Least Squares Discriminant Analysis (OPLS-DA), FDR= False Discovery Rate. The metabolites are categorized into fatty acids (FA) and amino acids (AA).

# Supplementary Table 3. Basic Information on Differential Metabolites between PCOS-NOR and PCOS-HOR

| **Metabolite** | **Type** | ***P* value** | **VIP** | **log2(FC)** | **FDR** |
| --- | --- | --- | --- | --- | --- |
| Glycine | AA | <.001 | 3.22 | -0.34 | <.001 |
| Cysteine | AA | <.001 | 2.84 | 0.47 | <.001 |
| Sarcosine | AA | <.001 | 2.35 | -0.33 | 0.01 |
| 5-methoxytryptamine | Try | <.001 | 2.00 | 0.70 | 0.05 |
| Succinic acid | TCA | <.001 | 2.32 | -0.23 | 0.05 |
| Tyrosine | AA | <.001 | 2.11 | -0.26 | 0.05 |
| beta-Alanine | AA | <.001 | 1.85 | -0.18 | 0.08 |
| Oxidized Glutathione | AA | 0.01 | 1.80 | 0.27 | 0.09 |
| Ornithine | AA | 0.01 | 1.83 | -0.30 | 0.09 |
| (10E,12Z)-Octadecadienoic acid(C18_2n-10) | FA | 0.01 | 1.42 | 0.39 | 0.09 |
| Fumaric acid | TCA | 0.01 | 1.74 | -0.37 | 0.09 |
| Alanine | AA | 0.01 | 1.90 | -0.16 | 0.12 |
| Valine | AA | 0.01 | 1.69 | -0.16 | 0.12 |
| Proline | AA | 0.02 | 1.99 | -0.20 | 0.17 |

Note: FC= Fold Change, VIP= Variable Importance in Projection value based on the Orthogonal Partial Least Squares Discriminant Analysis (OPLS-DA), FDR= False Discovery Rate. The metabolites are categorized into fatty acids (FA), tricarboxylic acid cycle (TCA), tryptamine(Try), and amino acids (AA).


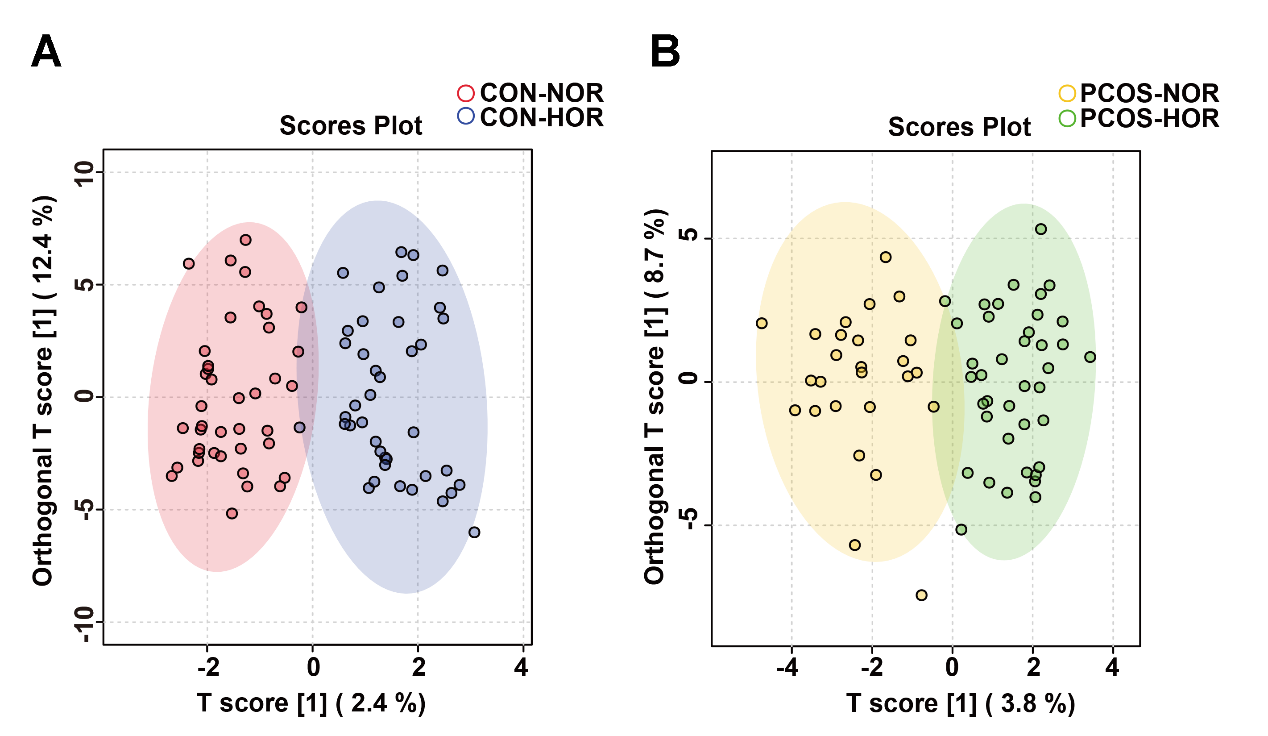


# Supplementary Fig 2. Subgroup Comparisons using Orthogonal Partial Least Squares Discriminant Analysis (OPLS-DA)

Each point on the plot corresponds to a sample, with the horizontal and vertical coordinates representing the values of the two factors that optimize discrimination. Various colors distinguish different groups, and the oval-marked area denotes the 95% confidence region of the sampling points.

# Supplementary Table 4. Binary Logistic Regression Analysis of Differential Metabolites: Adjusted OR (95% CIs) for HOR in the CON Group

| **metabolite** | **β** | **SE** | **Z** | **OR** | **95% CI** | ***P* value** |
| --- | --- | --- | --- | --- | --- | --- |
| 10-Pentadecenoic acid | 0.15 | 0.06 | 2.33 | 1.16 | (1.02, 1.13) | **0.020** |
| Glycine | -0.09 | 0.06 | -1.54 | 0.92 | (0.82, 1.02) | 0.124 |
| Oxidized Glutathione | 0.89 | 0.72 | 1.24 | 2.43 | (0.06, 9.92) | 0.215 |
| N-Acetyl-L-leucine | -41.04 | 29.26 | -1.40 | 0.00 | (0.00, 40.00) | 0.161 |

Note: SE = Standard Error, OR = Odds Ratio, CI = Confidence Interval. The regression models were adjusted for age (years), body mass index (BMI) (kg/m²), and anti-Müllerian hormone (AMH). Bold fonts indicate a *P* value < 0.05.

# Supplementary Table 5. Binary Logistic Regression Analysis of Differential Metabolites: Adjusted OR (95% CIs) for HOR in the PCOS Group

| **metabolite** | **β** | **SE** | **Z** | **OR** | **95% CI** | ***P* value** |
| --- | --- | --- | --- | --- | --- | --- |
| Glycine | -0.03 | 0.03 | -1.06 | 0.97 | (0.92, 1.03) | 0.288 |
| Cysteine | 0.02 | 0.010 | 2.35 | 1.02 | (1.01, 1.05) | **0.019** |
| 5-methoxytryptamine | 1.43 | 0.50 | 2.87 | 4.17 | (1.58, 11.06) | **0.004** |
| Sarcosine | -4.50 | 4.09 | -1.10 | 0.01 | (0.00, 33.46) | 0.271 |

Note: SE = Standard Error, OR = Odds Ratio, CI = Confidence Interval. The regression models were adjusted for age (years), body mass index (BMI) (kg/m²), and anti-Müllerian hormone (AMH). Bold fonts indicate a *P* value < 0.05.

# Supplementary Table 6. Spearman Correlation Analysis of Differential Metabolites and Clinical Characteristics in the CON Group (Coefficients).

|  | **10-Pentadecenoic acid** | **Glycine** | **Oxidized Glutathione** | **N-Acetyl-L-leucine** |
| --- | --- | --- | --- | --- |
| Age | 0.218 | 0.231 | -0.092 | -0.083 |
| BMI | -0.014 | 0.038 | 0.073 | -0.089 |
| Basal AMH | 0.160 | -0.277 | 0.322 | -0.177 |
| E2 on hCG day | 0.249 | -0.486 | 0.372 | -0.351 |
| Number of follicles ≥ 14 mm in diameter on hCG day | 0.207 | -0.376 | 0.397 | -0.304 |
| Number of follicles ≥ 17 mm in diameter on hCG day | 0.234 | -0.386 | 0.406 | -0.290 |
| Total dosage of Gn (IU) | -0.128 | 0.060 | -0.185 | 0.017 |
| Number of follicles | 0.107 | -0.337 | 0.336 | -0.284 |
| Follicle diameter | 0.094 | -0.335 | 0.127 | -0.072 |
| Number of oocytes retrieved | 0.245 | -0.339 | 0.393 | -0.250 |
| Number of mature oocytes | 0.137 | -0.287 | 0.321 | -0.197 |
| Number of MII oocytes | 0.240 | -0.357 | 0.402 | -0.241 |

# Supplementary Table 7. Spearman Correlation Analysis of Differential Metabolites and Clinical Characteristics in the CON Group (*P* value)

|  | **10-Pentadecenoic acid** | **Glycine** | **Oxidized Glutathione** | **N-Acetyl-L-leucine** |
| --- | --- | --- | --- | --- |
| Age | 0.052 | **0.039** | 0.415 | 0.466 |
| BMI | 0.905 | 0.740 | 0.522 | 0.430 |
| Basal AMH | 0.156 | **0.013** | **0.004** | 0.116 |
| E2 on hCG day | **0.026** | **<.001** | **<.001** | **<.001** |
| Number of follicles ≥ 14 mm in diameter on hCG day | 0.066 | **0.001** | **<.001** | **0.006** |
| Number of follicles ≥ 17 mm in diameter on hCG day | **0.036** | **<.001** | **<.001** | **0.009** |
| Total dosage of Gn (IU) | 0.258 | 0.597 | 0.101 | 0.883 |
| Number of follicles | 0.346 | **0.002** | **0.002** | **0.011** |
| Follicle diameter | 0.404 | **0.002** | 0.263 | 0.523 |
| Number of oocytes retrieved | **0.028** | **0.002** | **<.001** | **0.025** |
| Number of mature oocytes | 0.226 | **0.010** | **0.004** | 0.079 |
| Number of MII oocytes | **0.032** | **0.001** | **<.001** | **0.032** |

Significance is denoted by bold font for *P* value < 0.05.

# Supplementary Table 8. Spearman Correlation Analysis of Differential Metabolites and Clinical Characteristics in the PCOS Group ( Coefficients).

|  | **Glycine** | **Cysteine** | **Sarcosine** | **5-methoxytryptamine** | **Succinic acid** | **Tyrosine** | **beta-Alanine** | **Oxidized Glutathione** | **Ornithine** | **(10E,12Z)-Octadecadienoic acid(C18_2n-10)** | **Fumaric acid** | **Alanine** | **Valine** | **Proline** |
| --- | --- | --- | --- | --- | --- | --- | --- | --- | --- | --- | --- | --- | --- | --- |
| Age | 0.109 | 0.039 | 0.177 | 0.320 | -0.021 | -0.014 | 0.100 | -0.126 | 0.031 | 0.110 | -0.172 | 0.078 | 0.099 | 0.168 |
| BMI | 0.201 | 0.040 | 0.032 | -0.001 | 0.010 | 0.264 | 0.243 | -0.056 | 0.387 | 0.127 | -0.003 | 0.453 | 0.248 | 0.342 |
| Basal AMH | -0.257 | 0.113 | -0.228 | 0.054 | -0.033 | -0.020 | -0.048 | 0.294 | -0.046 | 0.074 | -0.046 | -0.048 | 0.084 | -0.216 |
| E2 on hCG day | -0.509 | 0.323 | -0.528 | 0.215 | -0.363 | -0.167 | -0.393 | 0.301 | -0.391 | 0.268 | -0.339 | -0.495 | -0.252 | -0.291 |
| Number of follicles ≥ 14 mm in diameter on hCG day | -0.481 | 0.418 | -0.487 | 0.207 | -0.398 | -0.115 | -0.254 | 0.352 | -0.284 | 0.216 | -0.276 | -0.443 | -0.097 | -0.292 |
| Number of follicles ≥ 17 mm in diameter on hCG day | -0.319 | 0.293 | -0.454 | 0.213 | -0.252 | -0.133 | -0.261 | 0.292 | -0.293 | 0.115 | -0.116 | -0.366 | -0.136 | -0.402 |
| Total dosage of Gn (IU) | 0.192 | 0.054 | -0.087 | 0.114 | -0.109 | 0.087 | 0.014 | -0.017 | 0.179 | 0.136 | -0.198 | 0.299 | 0.070 | 0.349 |
| Number of follicles | -0.408 | 0.425 | -0.327 | 0.217 | -0.331 | -0.037 | -0.222 | 0.378 | -0.168 | 0.215 | -0.236 | -0.304 | -0.076 | -0.184 |
| Follicle diameter | -0.056 | 0.060 | -0.216 | 0.021 | -0.028 | -0.082 | -0.085 | 0.021 | -0.159 | 0.020 | 0.103 | -0.048 | -0.112 | -0.189 |
| Number of oocytes retrieved | -0.471 | 0.369 | -0.439 | 0.297 | -0.405 | -0.266 | -0.296 | 0.332 | -0.326 | 0.192 | -0.261 | -0.311 | -0.204 | -0.276 |
| Number of mature oocytes | -0.370 | 0.296 | -0.382 | 0.178 | -0.348 | -0.256 | -0.235 | 0.270 | -0.386 | 0.140 | -0.250 | -0.247 | -0.168 | -0.226 |
| Number of MII oocytes | -0.479 | 0.377 | -0.445 | 0.299 | -0.423 | -0.261 | -0.300 | 0.323 | -0.335 | 0.210 | -0.264 | -0.309 | -0.216 | -0.282 |

# Supplementary Table 9. Spearman Correlation Analysis of Differential Metabolites and Clinical Characteristics in the PCOS Group (*P* value).

|  | **Glycine** | **Cysteine** | **Sarcosine** | **5-methoxytryptamine** | **Succinic acid** | **Tyrosine** | **beta-Alanine** | **Oxidized Glutathione** | **Ornithine** | **(10E,12Z)-Octadecadienoic acid(C18_2n-10)** | **Fumaric acid** | **Alanine** | **Valine** | **Proline** |
| --- | --- | --- | --- | --- | --- | --- | --- | --- | --- | --- | --- | --- | --- | --- |
| Age | 0.389 | 0.757 | 0.159 | **0.009** | 0.867 | 0.914 | 0.430 | 0.318 | 0.807 | 0.383 | 0.172 | 0.539 | 0.431 | 0.180 |
| BMI | 0.109 | 0.751 | 0.797 | 0.992 | 0.939 | **0.034** | 0.052 | 0.657 | **0.001** | 0.315 | 0.984 | **<.001** | **0.047** | **0.005** |
| Basal AMH | **0.039** | 0.370 | 0.068 | 0.667 | 0.796 | 0.874 | 0.703 | **0.018** | 0.714 | 0.556 | 0.717 | 0.703 | 0.503 | 0.084 |
| E2 on hCG day | **<.001** | **0.009** | **<.001** | 0.085 | **0.003** | 0.184 | **0.001** | **0.015** | **0.001** | **0.031** | **0.006** | **<.001** | **0.043** | **0.019** |
| Number of follicles ≥ 14 mm in diameter on hCG day | **<.001** | **0.001** | **<.001** | 0.098 | **0.001** | 0.363 | **0.041** | **0.004** | **0.022** | 0.084 | **0.026** | **<.001** | 0.441 | **0.018** |
| Number of follicles ≥ 17 mm in diameter on hCG day | **0.010** | **0.018** | **<.001** | 0.089 | **0.043** | 0.291 | **0.035** | **0.018** | **0.018** | 0.362 | 0.358 | **0.003** | 0.282 | **0.001** |
| Total dosage of Gn (IU) | 0.126 | 0.672 | 0.491 | 0.365 | 0.389 | 0.491 | 0.910 | 0.896 | 0.153 | 0.280 | 0.114 | **0.015** | 0.579 | **0.004** |
| Number of Follicles retrieved | **0.001** | **<.001** | **0.008** | 0.082 | **0.007** | 0.768 | 0.076 | **0.002** | 0.181 | 0.085 | 0.058 | **0.014** | 0.545 | 0.143 |
| follicle diameter | 0.656 | 0.636 | 0.084 | 0.871 | 0.827 | 0.514 | 0.501 | 0.866 | 0.205 | 0.875 | 0.413 | 0.704 | 0.373 | 0.131 |
| Number of oocytes retrieved | **<.001** | **0.002** | **<.001** | **0.016** | **0.001** | **0.032** | **0.017** | **0.007** | **0.008** | 0.125 | **0.036** | **0.012** | 0.104 | **0.026** |
| Number of mature oocytes | **0.002** | **0.017** | **0.002** | 0.155 | **0.004** | **0.039** | 0.060 | **0.030** | **0.002** | 0.268 | **0.045** | **0.047** | 0.182 | 0.070 |
| Number of MII oocytes | **<.001** | **0.002** | **<.001** | **0.016** | **<.001** | **0.036** | **0.015** | **0.009** | **0.006** | 0.093 | **0.034** | **0.012** | 0.084 | **0.023** |

Significance is denoted by bold font for *P* value < 0.05.

# Supplementary Table 10. Basic Information on Differential Metabolites between CON-NOR and PCOS

| **Metabolite** | **Type** | ***P* value** | **VIP** | **log2(FC)** | **FDR** |
| --- | --- | --- | --- | --- | --- |
| Pyroglutamic acid | AA | <.001 | 2.10 | 0.69 | <.001 |
| Indole | Try | <.001 | 2.16 | 0.52 | <.001 |
| Phenol | Organic | <.001 | 1.97 | 0.29 | <.001 |
| Sarcosine | AA | <.001 | 2.40 | -0.30 | <.001 |
| Threonine | AA | <.001 | 1.88 | 0.39 | <.001 |
| Aspartic acid | AA | <.001 | 1.76 | 0.33 | 0.01 |
| Oxidized Glutathione | AA | <.001 | 1.87 | 0.29 | 0.01 |
| 5-methoxytryptamine | Try | <.001 | 2.11 | -0.06 | 0.01 |
| Glutamic acid | AA | <.001 | 1.50 | 0.39 | 0.01 |
| Tryptamine | Try | <.001 | 1.91 | -0.25 | 0.01 |
| Tryptophol | Try | <.001 | 1.83 | -0.26 | 0.01 |
| Glutamine | AA | <.001 | 1.74 | 0.65 | 0.01 |
| Picolinic acid | Try | <.001 | 1.77 | -0.42 | 0.02 |
| Theanine | AA | <.001 | 1.58 | 0.21 | 0.03 |
| Lysine | AA | <.001 | 1.32 | 0.22 | 0.03 |
| Cysteine | AA | <.001 | 1.25 | 0.33 | 0.03 |
| Myristic acid (C14_0) | FA | 0.01 | 1.81 | 0.43 | 0.04 |
| 10-Pentadecenoic acid | FA | 0.01 | 1.41 | 0.12 | 0.04 |
| N-Acetyl-L-alanine | AA | 0.01 | 1.34 | 0.19 | 0.05 |
| Oxidized Glutathione | AA | 0.01 | 1.06 | 0.19 | 0.05 |
| Lignoceric acid (C24_0) | FA | 0.01 | 1.71 | 0.49 | 0.05 |
| Glycine | AA | 0.01 | 1.74 | -0.16 | 0.07 |
| Decane | Alkane | 0.02 | 1.02 | -0.05 | 0.11 |
| Benzenepropanoic acid | Other | 0.02 | 1.69 | 0.24 | 0.12 |
| Nervonic acid (C24_1n-9c) | FA | 0.04 | 1.10 | 0.38 | 0.16 |
| Malic acid | TCA | 0.04 | 1.41 | 0.55 | 0.16 |
| Citraconic acid | TCA | 0.04 | 1.46 | -0.12 | 0.16 |
| Pentadecanoic acid | FA | 0.04 | 1.10 | 0.32 | 0.17 |
| Malic acid | TCA | 0.05 | 1.37 | 0.56 | 0.19 |
| Hexanoic acid (C6_0) | FA | 0.05 | 1.22 | 0.83 | 0.19 |

Note: FC= Fold Change, VIP= Variable Importance in Projection value based on the Orthogonal Partial Least Squares Discriminant Analysis (OPLS-DA), FDR= False Discovery Rate. The metabolites are categorized into fatty acids (FA), tricarboxylic acid cycle (TCA), tryptamine(Try), and amino acids (AA)


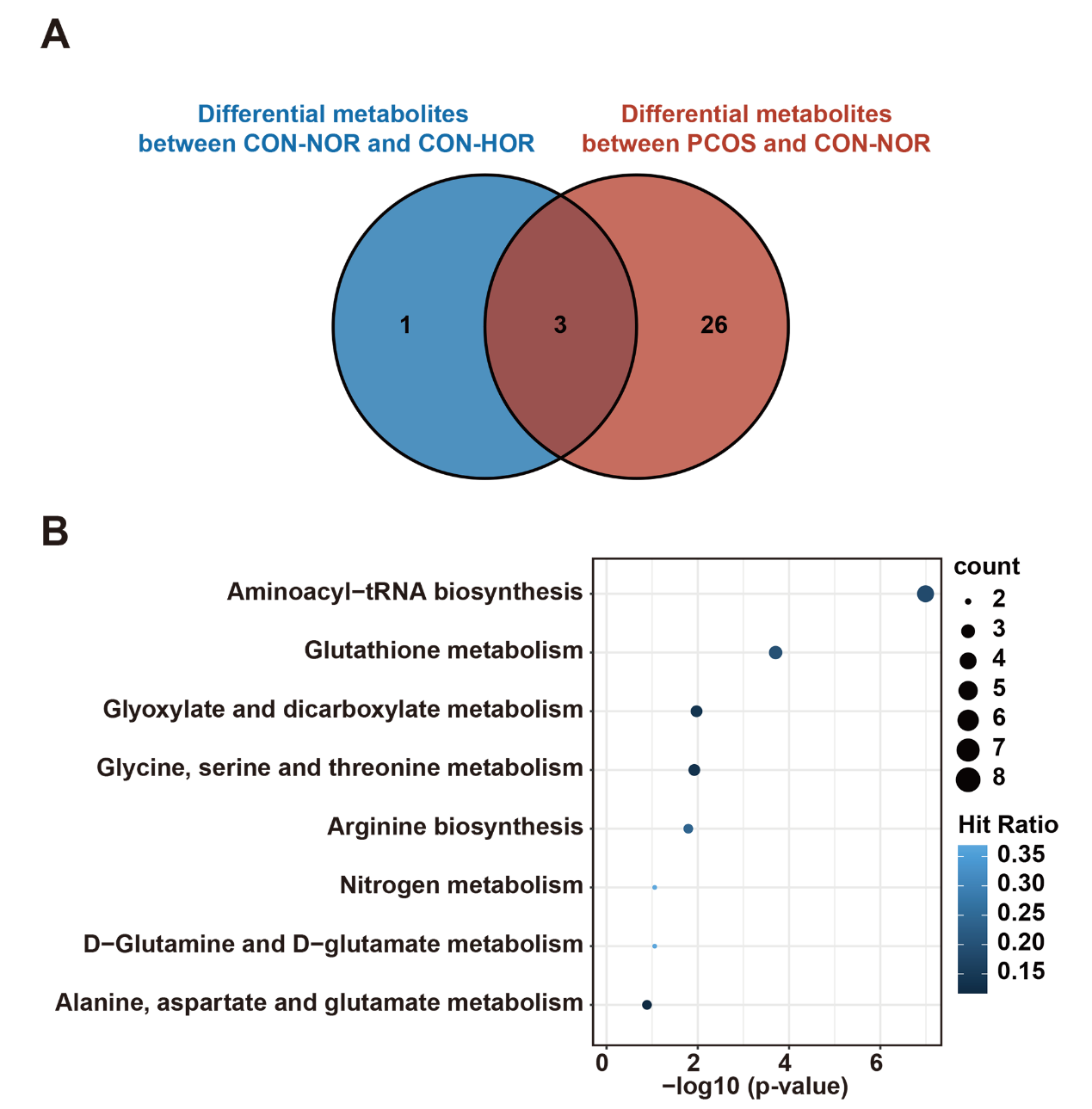


# Supplementary Fig 3. Integrated Metabolomic Analysis of PCOS and CON Subgroups

A. In the Wayne diagram, blue circles represent differential metabolites compared within subgroups of the CON group, red circles depict differential metabolites compared with both CON-NOR and PCOS groups. The overlapping region quantifies the degree of shared differentiation between these two sets of metabolites.

B. KEGG pathway enrichment analysis of differential metabolites identified in the comparison between the CON-NOR and PCOS groups. The vertical axis represents distinct metabolic pathways, and the horizontal axis indicates the Holm-adjusted *P* value. The circle size reflects the number of Hits, and the color indicates Hits. Ratio.
